# Retinal relaxation following membrane peeling: Effect on vision, central macular thickness, and vector analysis of motion

**Published:** 2020-05-12

**Authors:** Marc D. de Smet, Karina Julian, Jerick Maurin, Laurent P. Jolissaint, Marco Mura

**Affiliations:** ^1^Micro Invasive Ocular Surgery Center, Lausanne, Switzerland; ^2^Eye Institute, Cleveland Clinic Abu Dhabi, Abu Dhabi, United Arab Emirates; ^3^Department of Optical Instrumentation, University of Applied Sciences of Western Switzerland, Yverdon, Switzerland; ^4^Division of Vitreoretinal Surgery, King Khaled Eye Specialist Hospital, Riyadh, Saudi Arabia

**Keywords:** Epiretinal membrane, internal limiting membrane, optical coherence tomography, relaxation, scanning laser ophthalmoscopy, surgery, vector analysis

## Abstract

**Background:**

Epiretinal membranes (ERM) form as a result of an inward displacement of retinal structures. Removal of an ERM leads to the outward displacement of retinal vessels and visual improvement.

**Purpose:**

The aim of the study was to evaluate the direction and extent of displacement of retinal/superficial vascular structures after a membrane peeling procedure by means of image comparison and in selected cases, a vector analysis of displacement.

**Methodology:**

Scanning laser ophthalmoscope images of the retina of eyes undergoing ERM peeling were compared before and 6 months after surgery. Stratification was made between prominent and limited displacement, with assessment of visual acuity (VA), and central macular thickness (CMT). In three cases, using the optic nerve as reference, 50 landmarks were chosen within the posterior pole along large and small vascular structures allowing the construction of a vector map of displacement over 1 year.

**Results:**

Nine eyes with prominent and six with limited displacement were assessed. Improvement in VA was similar for both groups, while CMT drop was greatest for the worst group. Vector analysis showed that most vascular movement occurs over the first 6 months, covers most of the posterior retina, is centered around distinct nodes, and may lead to several hundred micrometers of displacement.

**Conclusions:**

Superficial retinal relaxation has no direct implication on visual recovery. It originates in nodes of retinal contraction. Its extent can be significant, covering most of the posterior pole.

**Relevance for Patients:**

A better understanding of retinal relaxation following the peeling of ERM may help better understand when intervention is required and which part of the membrane is critical to surgical success.

## 1. Introduction

Epiretinal membranes (ERM) form as a result of fibrocellular proliferation and extracellular matrix deposition at the vitreoretinal junction. These are associated temporally with the development of a posterior vitreous detachment [1]. As the ERM at the surface of the macula contracts, it is leads to blurred central vision, aniseikonia, and metamorphopsia [2-5]. Contraction at the surface of the retina leads to an increase in central macular thickness (CMT) and is associated with a decrease in best corrected acuity [5]. Using fundus images taken at timed intervals, Kofod and la Cour were able to demonstrate, using sectoral grids that tangential retinal vessel movement was correlated with worsening of vision, and that this was not a static but a dynamic process [5]. Similar results were observed using autofluorescence imaging [6].

Vitreomacular surgery releases these tractional forces, by eliminating points of attachment between the membrane and the retina and leading to visual improvement over several months [7-9]. On optical coherence tomography (OCT), lowering of CMT is observed, but a return to a completely normal contour with its characteristic foveal depression is seldom realized [8,10]. Pre-operative predictive signs of post-operative vision improvement remain difficult to establish. OCT characteristics in patients with ERM have been extensively studied but these have not been found so far to be predictive of visual outcome [11]. Based on autofluorescence images before and after vitreoretinal surgery, Nitta *et al*. suggested that if pre-operative hyperfluorescent lines present in a number of ERM eyes disappeared postoperatively, a better visual outcome was possible [12]. The observation was presumed to occur through the relaxation of retinal vessels, assuming their original position.

A number of studies have attempted to quantify the outward displacement of vessels following surgery using photographic images or scanning laser ophthalmoscopy (SLO) [5,8,13,14]. SLO provides a high spatial resolution of the retinal surface with an X-Y precision between 7 and 10 mm. If rotational aberrations are taken into account, it is possible to superimpose images taken at different time points. By comparing pre- and post-operative images taken in eyes undergoing macular peeling procedures for ERM, it is evident that retinal displacements vary greatly between eyes. Some vessels exhibit significant displacement while in other eyes, a limited shift is observed. This process is not only evident in perifoveal vessels, on close observation; it is also present in vessels located close to the vascular arcade. A commonly used technique in engineering and physical sciences is the use of vectors to indicate intensity and direction of a process of interest. We decided to apply this technique to vascular relaxation following macular pucker surgery. The aim of this study was to better understand the vascular relaxation process and to see if it correlated in any way with improvement in vision. We attempted to correlate the large or limited vascular shifts observed in patients with visual outcomes, and used vector matrices to quantify at multiple retinal vascular landmarks (50/eye) the direction and extent of vascular displacement over time. This approach confirmed that vascular displacement occurs over a large area. It defined a time course for this displacement extending over months, and as might be expected, displacement is greatest around the various nodes of fibrosis on the retinal surface.

## 2. Methodology

### 2.1. Patient selection and ethics approval

An electronic practice based database was retrospectively searched for patients, diagnosed with an ERM, having undergone an ERM peel with 6 or more months of follow-up and in whom spectral domain SLO-OCT images were available for analysis. Patients with prior ocular surgeries except in cases of cataract removal, or with additional macular pathologies were excluded from further analysis. Each patient signed an informed consent for the use of their anonymized medical data using a form approved by the medical ethics committee of the canton de Vaud, Switzerland. As this was a retrospective study using anonymized data, no further approval was required from the local ethics commission (http://cer-vd.ch/soumission/premiers-pas.html#c752; accessed January 12, 2020).

### 2.2. Data collection and surgical protocol

At each visit, as part of routine patient follow-up, a complete ocular exam was obtained including the measurement of best corrected visual acuity using a wall mounted Snellen chart, and the recording of a spectral domain SLO-OCT topography map centered on the fovea using a series D SLO-OCT Opko device (Tampa Florida, USA). For the purpose of the current analysis, the following data were recorded: sex, age at surgery, state of the lens, vision (pre-operative, post-operative at 1, 3, and 6 months, and when possible at 1 year), and CMT taken from the topographical map centered on the fovea at each of the above-mentioned time points.

Patients were operated by the same surgeon (MdS) under local anesthesia. Following a 25 G pars plana vitrectomy, the ERM was peeled over an area extending up to both temporal arcades. The surface of the retina was stained with either membrane or brilliant blue to confirm the removal of all membranes including the internal limiting membrane (ILM). If staining indicated the presence of the ILM, the ILM was removed from the foveal surface over a minimum area of three disk diameters.

### 2.3. Data analysis

Patients were stratified based on the degree of vascular displacement observed when the pre-operative SLO image taken from the topography map was superimposed on the image obtained 6 months following surgery. A significant shift was considered to have taken place if any of the vessels surrounding the fovea, including the vessels at the arcade had a displacement of more than 150 mm (the diameter of the temporal vein 2 mm proximal to its insertion into the optic nerve) [15,16]. Other patients were considered to have minimal change.

SLO images of three representative patients where further analyzed to determine the magnitude and direction of the retinal shift over time. A patient with a minimal shift and two patients with a significant retinal shift were selected. In one patient with a significant shift, there was a significant decrease in CMT while in the other there was not. For each time point, an SLO image centered on the fovea was chosen from the corresponding B-scan file in which there were minimal visible artifacts in scan lines, or obscuration of the image. Snapshot images of the appropriate scan were exported as tiff files. Statistical analysis was carried out using Prism (GraphPad Software, La Jolla, CA) version 5.0 d for MacOS. A non-parametric Mann–Whitney test was used to compare groups.

### 2.4. SLO image processing for vector analysis

#### 2.4.1. Preparing the raw SLO images for processing

The raw data were obtained from SLO images from the SLO/OCT scanning ophthalmoscope. The field of view was approximately 7 mm, centered on the macula. Only images showing the optical nerve were kept for further analysis. For each datapoint analysis, 20 images were compared and only appropriate images selected, and/or processed to minimize aberrations. Random horizontal shearing as part of the initial SLO image reconstruction procedure was identified by its unique signature, and when present images were discarded leading to the elimination of about 50% of images. Shadows in the selected raw images – due to eyelash or floaters in the vitreous humor – decreased the contrast in some part of the images. This was taken care of by limiting the pixel’s values to the range <image>±5*sigma (image), and by subtracting, from each image, its projection on a two-dimensional polynomial of order 4 (in x, y). The final images were normalized by their root mean square value. Then, the location of the center of the optical nerve on each image was identified by fitting a circle on the optical nerve edge, and all the images were superposed using the optical nerve center (ONC) as the reference point, with a (x, y) shift and some rotation, when needed. This averaging procedure increased the contrast of the vessels and decreased the amplitude of spurious structures. Images were ready for processing.

#### 2.4.2. Measurement of the retina post-surgery distortion over time

We used the displacement of the vessels’ junctions to build a vector distortion map across the retina. Measurement of the coordinate of the junctions was done by hand. On each image, we took about 50 reference points, distributed along the largest to the tiniest vessels. Note that because of the persistence of non-vascular features on our SLO images, in particular the pre-surgery shadows due to the retina puckering, it was not possible to use automated detection methods.

From a date to another, images did not have the same orientation in (x, y) and rotation, because the patient’s head was not systematically positioned the same way. To cancel this instrumental effect, which would have corrupted our vector field measurement, images were first numerically aligned in (x, y) with the ONC as the reference. Then, after measurement of the vector field, we looked for the rotation angle around the ONC that would minimize the mean vectors’ length across the whole field, our assumption being that over an area of 7 mm width centered on the macula, an overall shift or rotation of the retina between pre- and post-surgery is unlikely. We found a systematic rotation angle in the range 1-1.5 degrees, compatible with what was expected. We corrected the vector maps for this rotation. Finally, the vectors unit was transformed from pixels to millimeters on the retina, using a scaling factor measured on the images, assuming a 0.15 mm width for the largest vessels near the optical nerve [17].

## 3. Results

**Figure 1:**
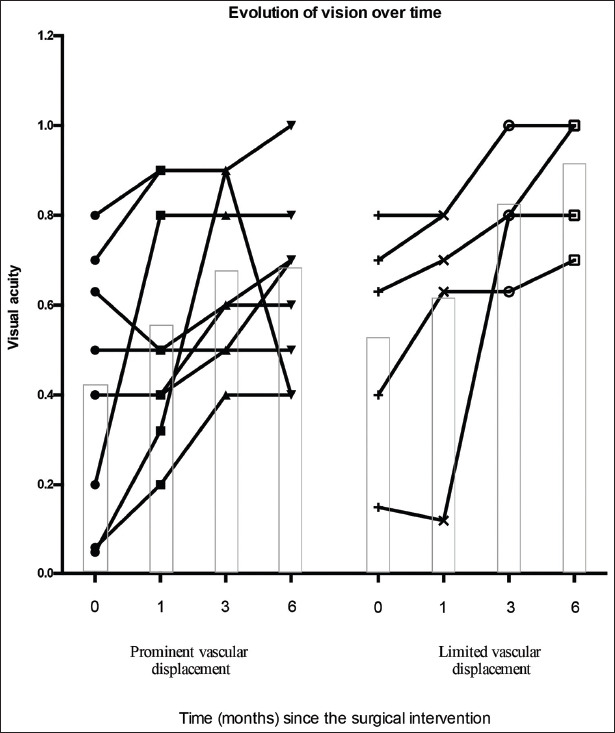
Evolution of vision over time. (Equivalent Snellen notation given as a decimal value). The height of the box plot corresponds to the average vision achieved at each time point.

A total of 15 eyes of 15 patients (nine males and six females), average age of 74 years (Standard deviation±7 years) met the inclusion criteria. Nine eyes had evidence of significant vascular displacement while six eyes showed little displacement. Vision improvement was noted in both groups of patients during the initial six post-operative months (Figure 1). Patients with prominent vascular displacement started with a lower average acuity (mean 0.4 [20/50]±0.3) compared to the group experiencing less displacement (mean 0.6 [20/30]±0.3), but this difference was not statistically significant. The rate of improvement in vision was comparable between the two groups over 6 months. In both groups, vision improved by a minimum of 3 lines on average. Improvement was more consistent in the patients experiencing less vascular displacement.

**Figure 2:**
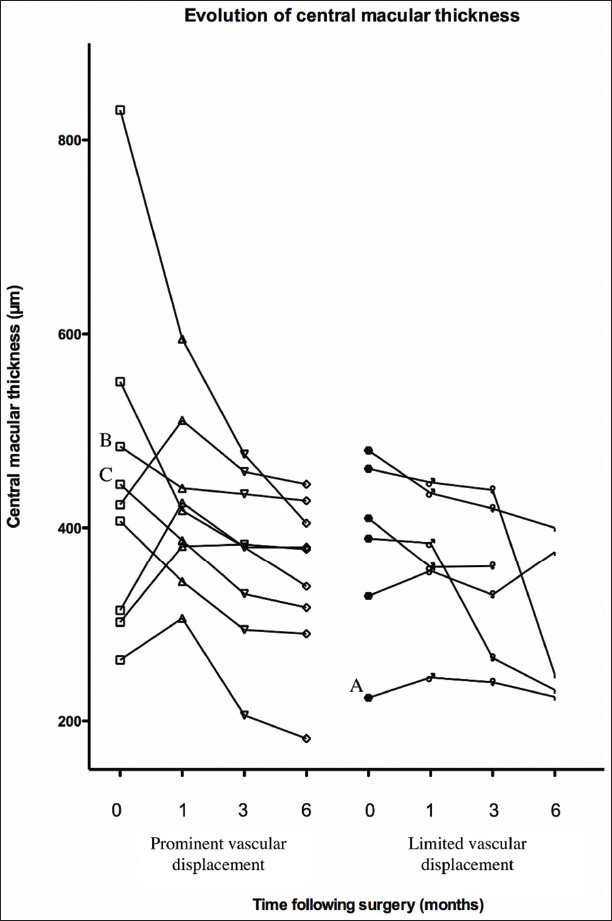
Evolution of the central macular thickness (μm) over time. The letters correspond to the patients in whom vector analysis was carried out in Figure 3.

Initial CMT was highest in the eyes experiencing prominent vascular displacement 424±171 mm (median 424 mm) versus 382±94 mm (median 400 mm), but this difference was not statistically significant. While CMT decreased in most eyes, in only one eye did it return to normal values (Figure 2). In none of the eyes, did the foveal contour return to normal. In eyes with prominent vascular displacement, the largest drop in CMT occurred in four of eight eyes within the 1^st^ month, and was present by 3 months in 7/8 eyes. While none of the eyes had macular edema before surgery, four eyes developed macular edema by 1 month after surgery associated with an increase in CMT. This edema when present took up to 8 weeks to resolve. In many cases, the fovea remained thickened even after complete resolution of the foveal fluid. In eyes with minimal displacement, macular edema was observed in only one of six eyes. While most of the reduction in CMT started shortly after surgery, the major drop in CMT started in some eyes as late as month 3.

**Figure 3:**
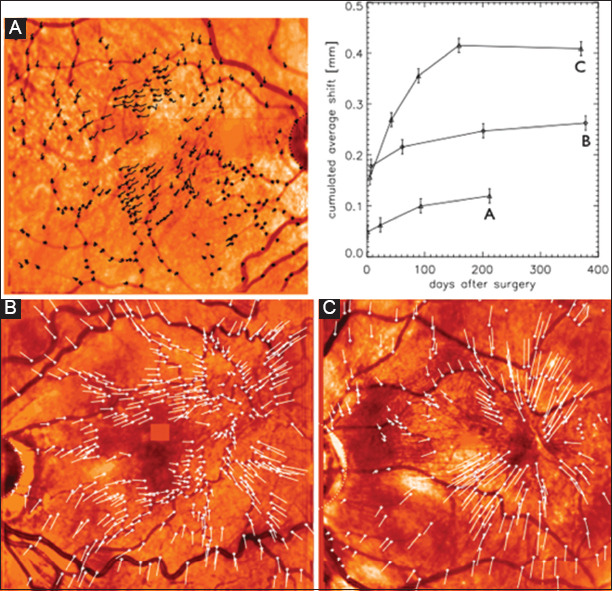
Vector representation of surface vascular movement in selected patients and graphical representation of cumulated average shifts over the analyzed time period per patient. (For each patient, the final image post-operative image was compared to the pre-operative scanning laser ophthalmoscopy image to generate the vector representations. Each line represents the displacement and direction of representative points).

**Figure 4:**
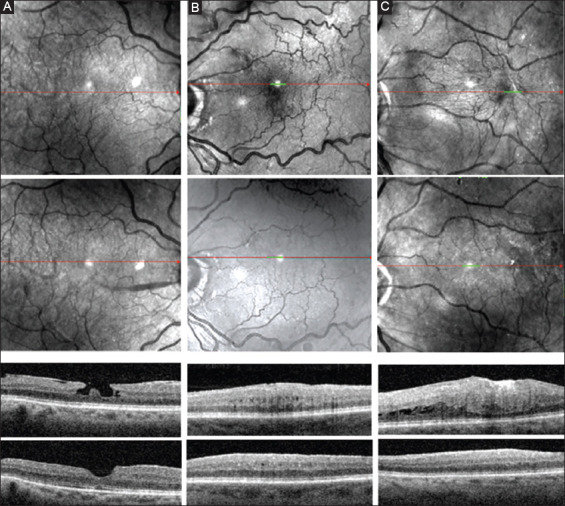
Scanning laser ophthalmoscopy and central optical coherence tomography scans take of patients studied in Figure 3. The upper SLO panels show the pre-operative scan, the lower SLO panels the last post-operative scan. In the OCT scan, the upper panel corresponds to a central macular scan before surgery, while the lower panel corresponds to the scan taken at the end of the study.

The vectors corresponding to the direction and degree of retinal relaxation are shown for the three selected cases in Figure 3. Vascular landmarks moved centrifugally away from the points of contraction. In case A, there were at least two sites of contraction but little outward displacement in the area of the fovea. On OCT, the traction was located around the fovea causing the formation of a pseudolamellar hole (Figure 4). This patient’s vision improved to 0.9 (20/25++) from 0.6 (20/30−). In case B, at least three foci of relaxation are seen (two temporal to the fovea and one inferonasal). On OCT, the fovea is thickened and is covered by a membrane. However, most of the outward displacement occurs at a distance from the foveal center. Outward displacement of vessels is noted at the arcade and beyond. Vision improved to 0.8 (20/25) from 0.6 (20/30−). While CMT decreased following surgery, most of the loss in height occurred temporal to the fovea and inferiorly, in the area corresponding to the vascular shift. In case C, the superficial contraction is located adjacent to the temporal edge of the fovea and is centered in a single focus. The outward shift extends to the arcade and is mainly located on the temporal side of the fovea. Vision improved from 0.6 (20/30−) to 1.0 (20/20) (Figure 4).

The cumulated average shift over time is depicted in Figure 3. The shift is greatest in the initial few months but continues for the length of follow-up in all three patients. In cases B and C, measurements were also obtained at 1 year post-surgery. Further relaxation was observed in patient B, but was not observed in patient C, the magnitude of this further relaxation was significantly lower than in the initial 6 months.

## 4. Discussion

Peeling ERM leads to a release of centripetal tractional forces and allows the retina to regain a more normal position. Several attempts have been made to quantify such movements using retinal landmarks based on retinal photography, SLO imaging, or fundus autofluorescence [5,6,8,13,14]. Using large vascular landmarks, it is possible to measure the general outward motion of the retina, comparing the size of pre- and post-operative surface areas bound by major vessels or landmarks [6]. Outward displacement of arterioles and venules from the foveal center or changes in the length of vessels between two branching points can also be determined and quantified per quadrant [14]. Major vascular shifts occurred in a temporal rather than nasal direction, as might be expected given the physical limitation posed by the optic nerve. However, in some cases such as macular hole closures following the removal of an ERM and tamponade with gas, a nasal shift of the foveal center has been observed on OCT. Such shifts, averaging 150 μm, were not observed with spontaneous closures and were also not observed in the current study [18]. Scan laser ophthalmoscopes generate images with a high spatial resolution in the order of 10 μm in the XY plane [19]. Therefore, it is possible to analyze in each patient, images taken at different time points both qualitatively and quantitatively, provided that imaging artifacts caused by shearing, rotation, tilting, or magnification are eliminated. By averaging and normalizing 20 images taken on the same day, most aberrations can be eliminated. The use the optic nerve as a common reference point allows for the alignment of images taken on different days. Using the same reference area, it is possible to eliminate any rotational artifact. Having corrected for tilt, rotation and size, it is then possible to use vectors to determine the direction and degree of vascular shift over an extended area of the retinal surface. When plotted over time, the rate of change can be determined.

Extensive puckers were associated with a lower initial vision, and a lower visual outcome, though the degree of vision recovery over a 6 month period was comparable to patients with less extensive ERM. Limitations to vision recovery were related in a number of cases to the presence of macular edema, often absent in the 1^st^ month after surgery, but appearing subsequently, as previously described [20]. This was also the origin of observed increases in retinal thickness, or the absence of a drop in thickness following surgery. It was observed equally in patients with prominent or limited vascular displacement following the removal of a membrane. Macular edema following membrane peeling has been reported in 1 to 10% of cases depending on the series [21]. Two distinct mechanisms are proposed. The first, as in Irvine Gass syndrome is related to the release of inflammatory mediators and is possibly related to the use of peripheral laser along the vitreous base [21]. Characteristically, it presents with outer retinal cystoid spaces. The second mechanism is related to pre-existing or surgically induced damage to the Muller cells and is characterized by intraretinal cyst formation not associated with fluorescein leakage, or with leakage visible in the very late stage. Both mechanisms, but predominantly the second, were observed in our patients.

The detailed analysis of vascular shifts at the retinal surface in three patients revealed that following surgery, relaxation takes place over an extended period of time. However, the bulk of the shift has occurred by 6 months. A study published in 2016, showed that the time course of improvement in metamorphosia following ERM peeling in patients followed over a 2 year period was most intense in the initial 6 months [22]. We observed that relaxation emanated from the points of fibrosis on the retinal surface and lead to a localized lowering in retinal thickness. The rate of relaxation appears to be variable. Additional cases would be necessary to determine whether this is dependent on the severity and extent of the superficial retinal distortions present before surgery. There appears to be limited or no correlation between retinal relaxation and improvement in vision. Indeed all three patients had the same improvement in vision though the variation in vascular shift was considerable. Vascular tortuosity and post-operative vascular shifts are reflective of changes at the retinal surface, while vision is more clearly related to changes in the ellipsoid zone and at the external limiting membrane [23-26].

Given our observations, one may question the need for extensive peels over the whole macular surface. Relieving traction at specific nodes of retinal fibrosis might eliminate traction and its deleterious effects on deeper retinal structures while avoiding the risk of damaging the nerve fiber layer [27]. Indeed a meta-analysis carried out in 2017, showed little difference in visual outcome with and without ILM peeling [28]. ILM peeling was associated with a lesser rate of recurrence, though an increase CMT was more frequently observed following an ILM peel.

As with all observational studies, its major limitation is the number of patients studied. To gain more insight on the characteristics of retinal relaxation following an epiretinal peel, it would be necessary to study a larger number of patients. Given the time involved in obtaining the measurements carried out in this study, automation of image acquisition and the determination of the vascular shifts at bifurcation points of retinal vessels would be particularly useful. With the advent of OCT angiography, it might also be of interest to study vascular shifts in perifoveal capillary beds, particularly at the level of the deeper capillary plexus as it may be reflective of the tractional forces exerted on the photoreceptor layer. Further studies may elucidate these questions.

## 5. Conclusions

A vector analysis of superficial vascular shifts has revealed the complex nature of retinal contraction and relaxation as a result of epiretinal membranes. The retina even beyond the arcades can be involved. Relaxation is present for up to 6 months after surgery. Vascular displacement of superficial vessels appears to lack correlation with vision improvement but a study of the inner retina, and possibly the outer capillary plexus may offer better correlation with the potential for vision improvement.

### Disclosures

None of the authors have any relevant disclosures in relation to this study.
